# Hedgehog Signaling during Appendage Development and Regeneration

**DOI:** 10.3390/genes6020417

**Published:** 2015-06-23

**Authors:** Bhairab N. Singh, Naoko Koyano-Nakagawa, Andrew Donaldson, Cyprian V. Weaver, Mary G. Garry, Daniel J. Garry

**Affiliations:** Lillehei Heart Institute, University of Minnesota, Minneapolis, MN 55455, USA; E-Mails: bnsingh@umn.edu (B.N.S.); koyano@umn.edu (N.K.-N.); andrewdonaldson2015@u.northwestern.edu (A.D.); cyprian@umn.edu (C.V.W.); garry002@umn.edu (M.G.G.)

**Keywords:** signaling pathways, hedgehog signaling, limb development, regeneration

## Abstract

Regulatory networks that govern embryonic development have been well defined. While a common hypothesis supports the notion that the embryonic regulatory cascades are reexpressed following injury and tissue regeneration, the mechanistic regulatory pathways that mediate the regenerative response in higher organisms remain undefined. Relative to mammals, lower vertebrates, including zebrafish and newts, have a tremendous regenerative capacity to repair and regenerate a number of organs including: appendages, retina, heart, jaw and nervous system. Elucidation of the pathways that govern regeneration in these lower organisms may provide cues that will enhance the capacity for the regeneration of mammalian organs. Signaling pathways, such as the hedgehog pathway, have been shown to play critical functions during development and during regeneration in lower organisms. These signaling pathways have been shown to modulate multiple processes including cellular origin, positional identity and cellular maturation. The present review will focus on the cellular and molecular regulation of the hedgehog (HH) signaling pathway and its interaction with other signaling factors during appendage development and regeneration.

## 1. Introduction

**Figure 1:**
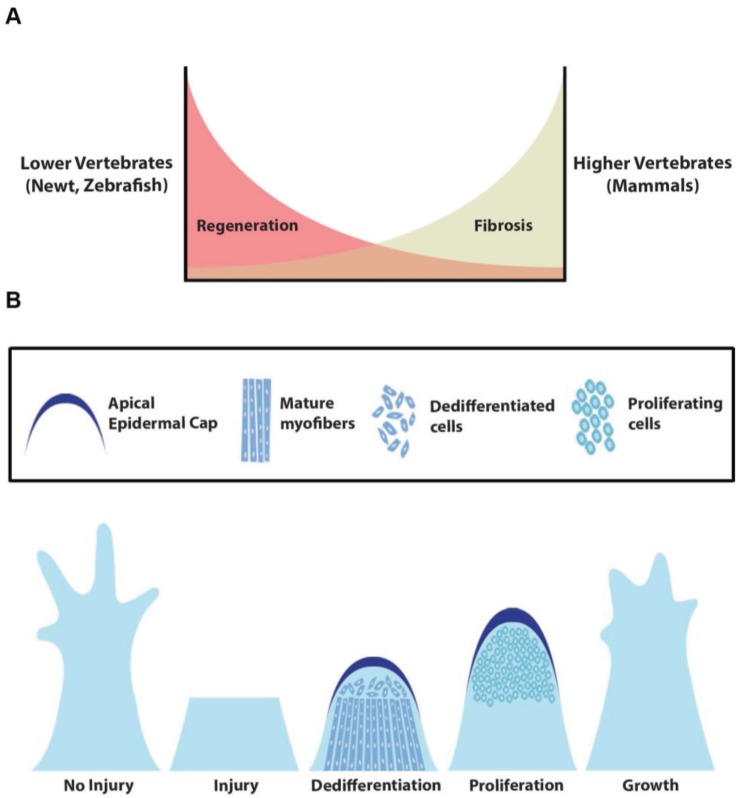
Regeneration and scar formation. (**A**) Lower vertebrates (newt and zebrafish) have tremendous potential to regenerate with minimal scar formation. In contrast, higher vertebrates (mammals) have a restricted regenerative ability marked by increased fibrosis and scar formation; (**B**) In the regenerative systems, tissue regeneration occurs in multiple steps including wound healing, dedifferentiation, proliferation, growth and patterning. Following injury, the epithelial cells proliferate to cover the injured area and form a multiple layered apical cap. Signaling from the regenerating regions initiate dedifferentiation of mature cells followed by proliferation, growth and redifferentiation. Note the key is in the upper panel.

Regenerative medicine holds tremendous promise for repair and restoration of damaged and/or diseased tissues. It is well recognized that there is considerable diversity regarding animal models and their capacity for regeneration (Table 1). Evolutionarily, the ability to regenerate appears to be inversely correlated with the complexity of an organism (Figure 1A) [1,2]. For example, mammalian models have a varied response to injury as some tissues have a tremendous capacity for regeneration (*i.e*., skin, blood, liver, skeletal muscle, *etc.*) and others are extremely limited (*i.e*., brain, spinal cord, heart, *etc.*) [3,4,5,6]. Those mammalian organ systems that have a more limited regenerative capacity typically respond to injury and/or chronic disease with a fibroproliferative response ultimately marked by avascular scar formation and/or fatty infiltration (Figure 1A) [1,6]. Molecular expressions profiling in lower organisms that have robust regenerative potential have identified factors that may govern this process [7,8]. Interestingly, loss of regenerative signals (FGF signaling) in regenerating organisms (*i.e*., zebrafish) results in scar formation and limited regeneration, suggesting a reciprocal relationship between regeneration and scar formation [9]. The varied regenerative responses between lower and higher organisms or between closely related mammalian tissues (*i.e.*, skeletal muscle *versus* heart) support the notion of either an active or dormant or absent molecular regenerative pathway and these pathways continue to receive intense interest.

**Table 1:** Differential regenerative potential in vertebrates.

Tissue	Species	Extent of Regeneration	Signaling Pathways
**Tail**			
	Mexican axolotl		WNT, BMP, NOTCH, SHH [8,10,11,12,13,14,15]
	(*Ambystoma mexicanum*)	Complete	
	Xenopus		
	(*Xenopus laevis*)		
	Larval Stage	Complete	
	Newt		
	(*Notophathalmus viridescencs*)	Complete	
**Limb**			
	Mexican axolotl		FGF, WNT, NOTCH, SHH, RA, BMP [16,17,18,19,20,21,22,23]
	(*Ambystoma mexicanum*)	Complete	
	Xenopus		
	(*Xenopus laevis*)		
	Larval Stage	Complete	
	Adult Stage	Spike (Incomplete)	
	Newt		
	(*Notophathalmus viridescencs*)	Complete	
**Fin**			
	Zebrafish (*Danio rerio*)	Complete	FGF, WNT, NOTCH, SHH, RA, BMP [17,24,25,26,27,28,29]
**Heart**			
	Newt		FGF, NOTCH, RA [6,9,13,30,31,32]
	(*Notophathalmus viridescencs*)	Complete	
	Zebrafish		
	(*Danio rerio*)	Complete	
	Mouse		
	(*Mus musculus*)	Complete	
	Neonatal Heart	Scar formation	
	Adult Heart		

Recent studies support the notion that tissue injury and the regenerative response are associated with the activation of embryonic/fetal gene regulatory pathways during regeneration [33,34]. Regeneration is marked by distinct stages of architectural restoration including: wound healing, blastema formation, cellular proliferation and differentiation (Figure 1B) [11,19,35,36,37,38]. Each of these stages has a specific molecular signature. For example, during the wound healing stage, programs involved in immunomodulation, cellular migration and extracellular matrix composition are expressed early following injury during the initial stages of regeneration (Figure 1B) [11,35,36,37]. Similarly, blastema formation and cellular proliferation phases are marked by cellular dedifferentiation and the activation of cell cycle regulatory genes (Figure 1B) [19,26,38,39]. The ability to form progenitor (or stem-like) cells from mature differentiated cell types (dedifferentiation) is a hallmark feature in lower regenerative organisms [7,19,26,38,39]. Dedifferentiation involves the loss of mature markers, changes in the chromatin status and nuclear architecture ultimately producing a cell that reenters the cell cycle [7,26,39]. Studies suggest that regenerative organisms such as newts and zebrafish have an inherent ability to induce dedifferentiation of the mature cells, thereby contributing to tissue regeneration [19,26,38,39,40] and may represent a key process/step that is relatively absent in higher vertebrates.

Although significant advancements have been made in this field, the factors which coordinate the growth, repair and regeneration of tissues are incompletely defined. Several signaling pathways including Hedgehog (HH), NOTCH, FGF and WNT pathways have been shown to regulate either one or multiple processes during regeneration (Table 1). Most of these pathways have been shown to regulate the proliferative, patterning and differentiation phases of the regenerative response [10,16,17,18,24]. In mammalian systems, the activation of the proliferative signals and suppression of differentiation signals in the terminally differentiated cells are two major barriers that may limit the regenerative response. Therefore, an enhanced understanding of the signaling pathways that regulate cellular proliferation and differentiation is essential for the field. This review explores the signaling and genetic networks that govern appendage regeneration in lower vertebrates recognizing that these data may ultimately contribute to an enhanced understanding of the regenerative response(s) in mammals.

## 2. Hedgehog Signaling Pathways during Limb Development

During embryogenesis, the limb bud develops from the proliferation of underlying mesenchymal cells that are in close approximation to the overlying ectodermal cells and is referred to as the apical ectodermal ridge (AER) [41,42,43]. Studies where the AER was removed from the developing limb bud of the chick resulted in the loss of limb bud outgrowth supporting the importance of the AER in limb growth [42,43,44]. Signaling molecules from the AER maintain the most distal cells in a proliferative state and promotes limb development [41,42,43,44,45,46].

Multiple signaling pathways are involved in regulating the limb bud development. For example, FGF, HH and WNT signaling coordinately function to maintain the developing limb bud [41,43,44,45,47,48]. The HH signaling cascade involves multiple molecular interactions operative in the transmission of the signal [41,49,50,51,52]. In vertebrates, three hedgehog (HH) proteins including Sonic hedgehog (SHH), Indian Hedgehog (IHH) and Desert Hedgehog (DHH) have been documented in mediating the HH signaling pathway [50,51]. The knockout of DHH has no phenotypic abnormalities [51,52,53]. In contrast, SHH- and IHH-null mice develop congenital abnormalities and lethality (Table 2) [54,55]. Each HH ligand is secreted and participates in a conserved HH signaling pathway [49,50,51]. The initiation of HH signaling occurs at the primary cilia [50,56]. Multiple studies have indicated that upon activation of HH signaling, Supressor of Fused (SuFu) proteins together with Gli (SuFu-Gli) are recruited to the cilia, facilitating the dissociation of the SuFu-Gli complex [56]. This results in the release of Gli proteins and further activation of HH signaling [56]. Several studies have indicated that ciliary proteins including intraflagellar transport proteins (IFT) are critical for activation and transduction of the HH signaling [50,56,57]. Importantly, it has been shown that mutation of the ciliary protein recapitulates HH signaling defects, suggesting a critical requirement of ciliary proteins in HH signal transduction [56,57]. For example, mutant mice for Dync2H1 and IFT144 proteins have been shown to be defective in skeletal morphogenesis, craniofacial defects and appendicular defects [56,57,58,59].

**Figure 2:**
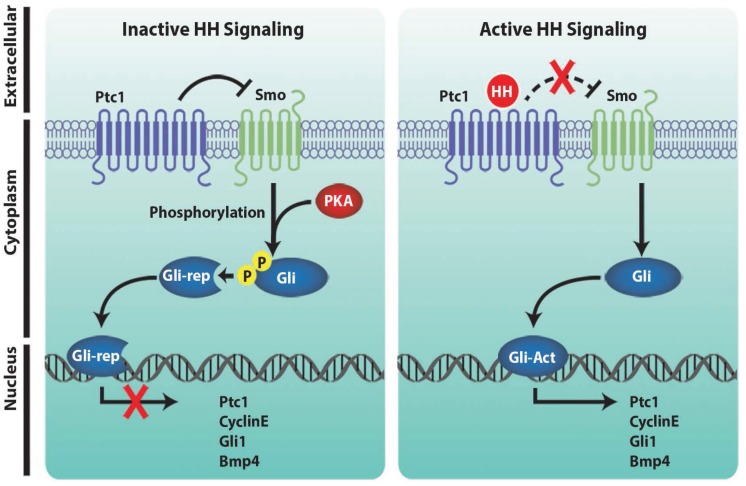
Schematic illustration of the HH signaling pathway. HH is a secreted morphogen which acts in an autocrine and paracrine fashion. In the absence of HH morphogen, Patched1 (Ptc1), a transmembrane protein, inhibits Smoothened (Smo) activity. Inhibition of Smo activity by Ptc1 has been hypothesized to involve a second messenger mediated mechanism. In the absence of Smo activity, protein kinase A (PKA) phosphorylates Gli proteins (a downstream target of HH signaling) leading to the generation of repressor Gli (Gli-rep), thereby resulting in inactive HH signaling. The binding of the HH morphogen results in loss of Ptc1 activity and subsequent activation of Smo activity. Activated Smo then transduces the signal, resulting in the activation of Gli2A (Gli-Act) and transcription of downstream targets.

In the absence of the HH morphogen, Patched 1 (Ptc1, a membrane protein) prevents the activation of HH pathway by inhibiting Smoothened (Smo) activity (Figure 2; inactive state) [49,50,51,52,60,61,62]. Upon binding of the HH morphogen, Ptc1 undergoes a conformational change, and is no longer able to suppress Smo activity. This results in an activation of the intracellular signal resulting in translocation of Gli proteins (Gli1, Gli2A and Gli3) into the nucleus (Figure 2, active state) [49,50,51]. Gli proteins are transcription factors which activate their downstream targets to modulate cell growth, proliferative and cell survival genes [51,60,61,63,64,65,66]. Elegant studies by Vokes *et al.* have demonstrated *Blimp1* as a direct downstream target of Gli factors [63]. Recent studies have implicated Gli-independent activation of HH signaling and categorized as non-canonical HH signaling [50,51]. In non-canonical HH signaling (non-Smo and non-Gli), HH activation results in disruption of the Ptc1-cyclin B1 complex and promotes the Ptc1 affinity to GRK2 [51]. Similarly, Gli-independent HH signaling has been shown to be involved in the regulation of the actin cytoskeleton by modulation of RhoA and Rac1 GTPases [51,60,64]. Both canonical and non-canonical HH pathways have been described in angiogenesis, development and tumorogenic processes [49,50,51,60,61]. The importance of the HH signaling pathway is evident from the genetic knockout studies, as gene disruption strategies of HH signaling members resulted in multiple developmental defects and lethality (Table 2).

**Table 2:** Genetic models and HH signaling during development.

Genotype	Lethality	Phenotype
**Shh-/-**	Embryonic lethality (E11.5–E18.5)	Midline structural defects Defective distal structure Dorsoventral patterning defects [41,54]
**Ihh-/-**	Partial embryonic Lethality	Skeletal defects Mesenchymal loss Chondrocyte proliferation defects [41,55]
**Smo-/-**	Embryonic lethality (E9.5–E10.5)	Midline structural defects Cardio-vascular defects L/R asymmetry defects [41,67,68]
**Shh-/-; Ihh-/-**	Embryonic lethality (E9.5–E10.5)	Midline structural defects Cardio-vascular defects Abnormal forebrain Patterning defects [41,64,67,68]
**Ptc1-/-**	Embryonic lethality (E9.5–E10.5)	Open neural tube defects Cardiac morphogenesis defects [69]
**Gli1-/-**	Viable	No obvious phenotype [66]
**Gli2-/-**	Embryonic lethality (E15.5–E18.5)	Defective lung outgrowth [66]
**Gli1-/-; Gli2-/-**	Embryonic lethality (E15.5–E18.5)	Defective growth and patterning of lung lobes Notochord regression defects Defective spinal cord ventral midline [66]
**Shh Morpholino (Zebrafish)**		Reduced myoseptum Defective somitic patterning Partial cylopia [70]
**Msx2-Cre;Smo^L/L^**	Lethality (birth)	Patterning defects [60,71]
**Prx1-Cre;Ptc^L/L^**	Viable	Patterning defects [41,69]

**Figure 3:**
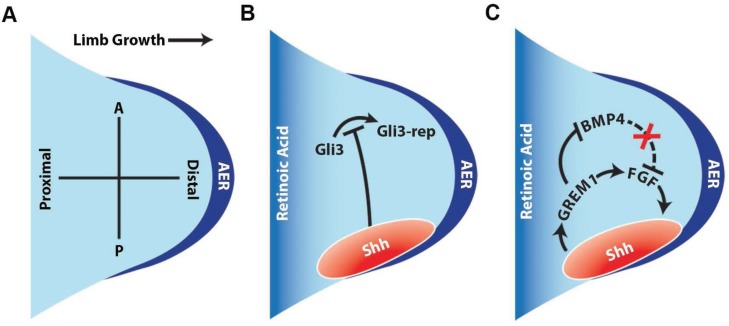
HH signaling and limb development. (**A**) Schematic outlining the different axises of the developing limb. Limb bud formation is initiated at the defined region of the embryonic axis. The proximal-distal (PD) axis is defined by the direction of the limb outgrowth and the antero-posterior (AP) axis is defined by the sequence of the digits 1 (thumb) to 5 (little finger); (**B**) In the developing limb bud, an apical ectodermal ridge (AER) is formed at the distal region of the bud. In the AER region, FGF signaling is initiated in a posterior-anterior fashion forming the AER-FGF zone. Retinoic acid (RA) signaling regulates the proximal development of the limb, whereas the distal region (progression zone) is controlled by multiple signaling factors. FGF signaling initiates the HH signaling in the posterior region of the limb bud and the expression of HH signaling is maintained by HOX genes, Tbx and Fgf8 expression. HH signaling inhibits the constitutive processing of Gli3 to its repressor form (Gli3-rep); (**C**) The posterior region contains high Gli3A and low Gli3-rep and reverse is observed in the anterior region. Following activation, Grem1 (BMP antagonism) functions are required to relay the HH signals to the AER to maintain FGF signaling, thus forming a HH-Grem1-FGF feedback loop in the developing limb bud.

The limb bud grows in a proximal-distal (PD) axis and patterning occurs from anterior-posterior (AP) axis (Figure 3A). Multiple reports demonstrated that FGF signaling between the AER and the underlying mesodermal cells function to coordinately generate the PD axis during limb development [41,42,43,44]. Genetic analysis revealed that Fgf8 from the AER and Fgf10 from the underlying mesenchymal cells are essential for limb growth [41,42]. In tetrapods, the development of forelimb and hindlimb position is specified by the T-box factors, Tbx5 and Tbx4. Both forelimb and hindlimb development are regulated by distinct signaling cascades [45,46,47]. For example, the regional expression of Fgf10 is regulated by Wnt2b in the forelimb and by Wnt8c in the hindlimb [48]. In contrast to the PD axis, the AP axis during limb development is modulated by SHH morphogens [41,65]. The expression of SHH is confined to the posterior region of the limb bud and has a graded expression pattern [72,73]. In the anterior region, SHH prevents the processing of Gli3 to form Gli3-rep, which functions as a repressor (Figure 3B) [41,49,64,66,73,74].

The genetic loss of *Shh* results in the absence of posterior digits (digits 2–5) without affecting the anterior digit (digit 1) [54,68,73]. Similarly, loss of *Ihh* leads to failure of osteoblast development and defective limb development [55,67]. The development of digit 1 depends on the expression of HOX, and Tbx5 factors [41,75,76,77]. Expression of SHH is initiated by Hoxb8 expressed in cells collectively referred to as the zone of polarizing activity (ZPA) and maintained by Fgf4 and Fgf8 from the AER [41,42,43,74,76,77,78,79]. Although each of the limb developmental processes has been shown to be distinctly regulated by a set of signals, increasing evidence supports the notion that these signaling cascades are closely interlinked and modulate the collective activity (Figure 3C) [74,77,80]. For example, experiments using soaked FGF beads showed that FGF maintains SHH expression in the AER deficient limb bud [78,79,80]. Similarly, Gremlin1 (Grem1) was shown to be required to relay SHH signals to the AER to promote FGF expression thereby defining a SHH-Grem1-FGF feedback loop [79,80] (Figure 3). This signaling loop was further confirmed by the genetic inactivation studies of Grem1 which led to the disruption of limb bud development and specification [78,79,80]. The expansion phase of limb growth is controlled by involvement of multiple signaling centers including ZPA-SHH, AER-FGF and WNT signaling during limb organogenesis [41,48,50,51,79,80]. During the later phases of limb bud development, the digit identity is dependent upon BMP activity. BMP signaling acts downstream of SHH signaling and facilitates the process of digit patterning [80,81]. It should be noted that BMP dependent signaling aids in the removal of webbing between the digits rather than digit identification [81]. Importantly, deciphering the key factors that govern limb formation has defined new molecular cues and linkage between the signaling pathways and transcription factors.

## 3. HH Signaling in Fin Regeneration

Urodele amphibians and teleost fish have an extraordinary capacity to regenerate injured appendages including tail, limb and fin [11,20,82]. A number of studies have demonstrated common regulatory networks in appendage development and regeneration [20,25,83]. Genetic as well as pharmacological studies have shown involvement of SHH, WNT and FGF signaling in the regulation of the proliferative response during appendage development [12,18,83]. As observed developmentally, these signaling factors and others such as Bmp2/4, Gremlin1, RA and NOTCH have also been shown to regulate the regenerative processes [11,21,80,84].

**Figure 4:**
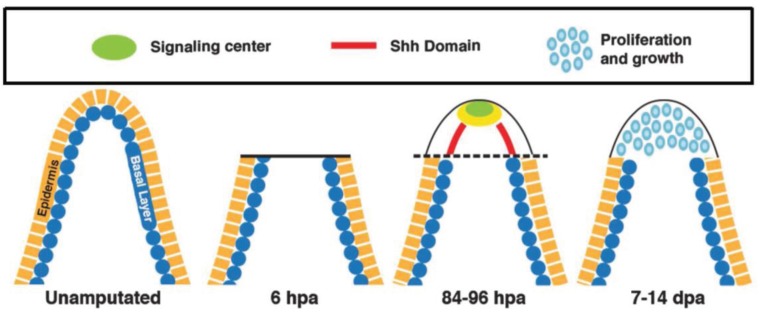
Sequence of regenerative events and HH signaling. Longitudinal section of an unamputated and regenerating fin ray showing the basal layer (blue) and outer epidermis (yellow). Epidermal cells cover the wound and mesenchymal cells from the stump proliferate and migrate distally to form the blastema. HH signaling is induced in the lateral basal epidermal layer and a signaling center (green) in the apical region (yellow) that includes: FGF, WNT and BMP signaling leads to the induction of cellular proliferation (blue) and regeneration. Note the key is in the upper panel.

Teleost fish have a tremendous capacity to regenerate a variety of tissues including heart, spinal cord and fin [27,28,85,86]. Upon amputation, fin regeneration occurs in three distinct stages including: wound healing, blastema formation and regeneration (regrowth) from the plane of amputation. Transcriptional profiling during fin regeneration revealed differential gene expression associated with these distinct stages in zebrafish [27,86]. These differential molecular programs included a number of developmental transcripts and signaling factors involved in regulating fin growth such as Fgf, Bmp2b, β-catenin, Shh, Hoxa11b and Hoxa13b [17,25,48,86]. In the wound epidermis, early expression of WNT signaling factors regulate the formation of the thickened epidermis termed the epidermal cap [17,25]. The proliferative response of the epidermal cells results in the formation of the thickened epidermal cap. Recently, Lee *et al.* described the existence of distinct types of cells beneath the epidermal layer which defines the regional expression domain for *Shh* signaling (Figure 4) [86]. FGF signaling in the adjacent layer helps localize and maintain *Shh* expression in the proximal region of the regenerating fin, whereas in the distal region, the expression of *Shh* is reduced by a Ras-Wnt5b signaling mechanism [25,86]. These findings suggest a critical role for epidermal signals in the control of the signaling domain during the regenerative response (Figure 4).

The zebrafish genome harbors five hedgehog genes namely Sonic hedgehog (*Shh*), Indian hedgehog (*Ihh*), Tiggywinkle hedgehog (*Twhh*), Echidna hedgehog (*Ehh*) and Desert hedgehog (*Dhh*). However, only *Shh*, *Ihh* and *Twhh* have been shown to participate in fin development and regeneration [87]. During fin regeneration, both *Shh* and *Ihh* are activated in the blastemal tissue and regulate blastemal proliferation, maintenance and tissue growth [25]. Laser-mediated ablation of *Shh* expressing cells during fin regeneration resulted in aberrant osteoblast differentiation and defective branching morphogenesis underscoring the importance of Shh signaling in fin regeneration [88]. Expression of Shh morphogens is induced in the lateral basal epidermal layer and they regulate expression of Bmp2b. Inhibition of either Shh signals or Bmp signaling resulted in loss of scleroblast differentiation and bone formation [25,29,86]. Interaction with other signaling pathways including Fgf and Wnt signaling with the Shh signaling pathway during fin regeneration has been shown to amplify the regenerative response [25,86,89]. Furthermore, studies have demonstrated expression of canonical Wnt signaling members such as Wnt5, Lef1 and beta-catenin in the wound epidermis during the regenerative process. It is noted that both Shh and Lef1 are expressed in a similar region of the regenerating fin tissue and inhibition of RA signaling or Fgf signaling results in loss of both Lef1 and Shh expression [25,86,88,89]. These studies indicated a common regulatory mechanism for both Shh and Wnt signaling centers.

Additional reports have indicated that WNT signaling acts downstream of SHH signaling as the activation of β-catenin could rescue SHH inhibition phenotypes [18]. These findings demonstrated that SHH and WNT pathways converge at a common node to regulate the regenerative process. Similar to SHH and WNT signaling, BMP signaling has also been shown to be involved in the development of osteoblasts and the maturation process [29]. Interestingly, ectopic induction of Bmp2b resulted in increased expression of osteoblast transcription factors required for differentiation [29]. Similar results were obtained following ectopic expression of SHH or BMP2b in the fin rays. These results supported the hypothesis that SHH activity was mediated via BMP signaling. The ectopic formation of bony tissue was due presumably to changes in the molecular signature of the responding cells or alternatively to a perturbation of the differentiation program that remains undefined [25,29]. A recent study by Knopf *et al.* has indicated that dedifferentiation of the osteoblast cells was a prerequisite for cellular proliferation [26]. These dedifferentiated cells proliferated in a FGF-dependent signaling mechanism. Multiple studies have established that SHH acts downstream of FGF signaling and regulates cell division and growth [21,25,82,86,89]. Whether tissue regeneration is governed solely by dedifferentiating cell types or whether precursors are mobilized from other tissues (transdifferentiation) is an area of intense research. Definition of the signaling factors that regulate the dedifferentiation process has yet to be defined, however it would be interesting to examine whether a combination of signaling factors could induce dedifferentiation in mature cell populations.

## 4. HH Signaling in Limb and Tail Regeneration

Urodele amphibians have a robust capacity to regenerate appendages in response to injury [13,16,40]. Similar to zebrafish fin regeneration, appendage (*i.e*., limb or tail) regeneration occurs in several steps including wound healing, dedifferentiation, proliferation, growth and patterning (Figure 1B) [18,24,40]. Animal models including the newt, axolotl and salamander can completely regenerate the amputated limb and heart over a 60 day period [13,14,18]. During limb and tail regeneration, the critical stages include the formation of the apical epidermal cap (AEC) and the formation of blastemal tissue [90]. Lineage tracing experiments have revealed dedifferentiation of the mature muscle fibers that contribute to the blastema during tail regeneration [91,92]. The importance of these structures is evident by the loss of regeneration with the removal of the AEC or blastemal tissue, thereby suggesting that these are critical structures required for regeneration [14]. Formation of the blastemal structure involves tissue histolysis and down regulation of differentiation markers. Interestingly, the extent of dedifferentiation differs considerably within the species during regeneration. For example, two salamander species (newt and axolotl) had marked differences in the dedifferentiation process; dedifferentiated cells contributed principally to the regenerating limb in the newt, whereas the resident Pax7^+^ cells were the main source of the regenerating tissue in the axolotl [18,93,94]. These studies support the hypothesis that the mechanism(s) as well as the cellular processes may be an inherent property of a given species. The dedifferentiated cells begin to proliferate at approximately 10–14 dpa and form a blastema, which then re-differentiates to restore the cellular architecture within a 6–9 week period [18,93,94] The cross-talk between signaling factors from neighboring tissues and their contribution to the regenerating blastema pose outstanding questions. Moreover, it is unclear which cells contribute to the regenerating limb tissue as it involves a continuous growth of multiple tissues including muscle, blood vessels, bone and connective tissue. A recent study by Kragl *et al.* (2009) demonstrated that progenitor cells during limb regeneration were derivatives from the respective tissues with restricted potential to contribute only toward the regeneration of specific lineages [95]. Other appendages such as the tail could regenerate completely in 14–21 days and involves similar stages of regeneration as that of fin and limb regeneration [15]. These results suggest that although the timing for regeneration of different tissues vary, the central pathways and processes are commonly shared. Therefore, the identification of factors or signals from these regenerating tissues may provide new insights regarding regenerative therapies.

**Figure 5:**
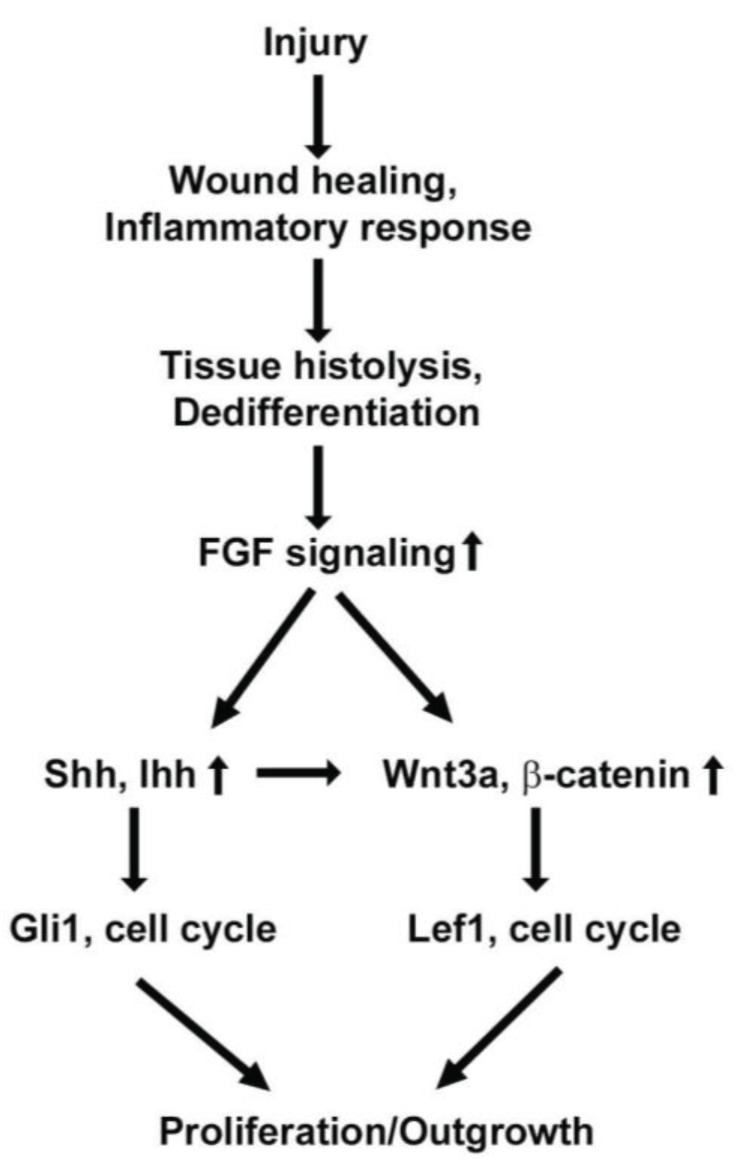
Hierarchical signaling pathway expression regulates regeneration. Tissue injury results in the activation of the inflammatory response at the site of damage. Following inflammation, lower vertebrate regeneration requires cellular dedifferentiation and cellular proliferation. FGF signaling is initiated at the early stages of regeneration, which further activates HH signaling. Both FGF as well as HH signaling pathways directly and indirectly activate WNT signaling. These factors activate the genes required for cell cycle and growth during regeneration.

Genome wide expression analysis during appendage regeneration has indicated enrichment of early and late genes [92]. Early enriched transcripts include genes expressed in wound epidermis, peripheral nerves and mesenchymal tissue, whereas late genes have included transcripts regulating cellular proliferation and growth [8]. Importantly, the signaling pathways seem to be common in both early and late gene expression analysis. Multiple studies focused on appendage development and regeneration support the notion that common signaling pathways including: FGF, HH, WNT and BMP are activated both during development and are reactivated during lineage regeneration [18,22,41,96]. Specifically, FGF, WNT and HH signaling have been well documented and their hierarchical relationships have been defined in detail in the regenerating appendages (Figure 5) [18,41,96]. An elegant study by Lin and Slack have demonstrated that WNT signaling acts downstream of FGF signaling during tail regeneration [12]. Similarly, Tgf-beta signaling has been documented in regulating wound epidermis and proliferation during tail regeneration. Studies suggest that FGF signaling modulates expression of HH signals which play a critical role in proliferation, growth and patterning [18,20,25,82,89]. As observed during limb bud development, HH factors are expressed early in the posterior region of the blastemal tissue, that serves as a signaling center for anterior-posterior (AP) patterning [18,83]. We and others have demonstrated that the HH signal is essential for limb regeneration and required in a spatial and temporal fashion during regeneration [18]. Early inhibition of HH signaling resulted in patterning defects whereas later inhibition led to reduced growth without patterning defects [18]. Similarly, the activation of HH signaling in the xenopus froglet resulted in induction of patterning events, which is otherwise absent in the froglets and results in spike formation [22]. In addition to the patterning, HH signaling modulated the Pax7^+^ (muscle progenitors) cell population and the regenerating fibers of the growing limb tissue [18]. These progenitor cell populations serve as a stable source of reserve cells for multiple rounds of limb regeneration [18,93,94]. These findings further indicated that HH signaling modulates different targets at multiple stages during regeneration. Several lines of evidence suggest that inhibition of HH signaling has a profound effect on the proliferative capacity of tissues, thereby indicating that it has a central role in activating proliferative signals during regeneration [15,18].

Canonical HH signaling activation occurs through Smo-Gli mediated pathways and Cyclind2, Cyclinb and Cycline1 have been shown to be authentic downstream targets (Figure 5) [96]. This would suggest that HH signaling activates proliferation by modulating these factors. Alternatively, another downstream effector of HH signaling during regeneration is Bmp2; however, BMP signaling could modulate regeneration in a dependent and/or independent HH signaling mechanism [25]. Regulation of *Shh* expression is modulated by the methylation status and epigenetic modification of its enhancer region but not at the promoter region [23]. It has been shown that in the regenerating state, hypomethylation of the *Shh* enhancer resulted in its expression in the regenerating limb bud [23]. In contrast, in the non-regenerating state, the enhancer region remains highly methylated (hypermethylation) which results in regeneration failure and patterning defects.

## 5. Concluding Remarks

Signaling factors have an essential role in regenerative biology. It is important to decipher the common regulatory networks and their interactions to define the overall regenerative blueprint or regenerative map that will serve as a platform for regenerative therapies. Recent studies have indicated that activation of HH signaling is critical for liver regeneration in mammals, as inhibition of HH signaling resulted in inhibition of hepatocyte proliferation, progenitor response and matrix remodelling [97]. These findings support the notion that distinct signaling pathways that govern regeneration in lower organisms may also promote regeneration in mammals. The role of HH signaling in tissue growth and patterning during development is well documented, however, its role in tissue regeneration is less clear. Also, emerging areas of investigation include the definition of the upstream regulators of the HH signaling pathway during development and regeneration; the definition of all the downstream targets of the HH signaling pathway (*i.e*., Gli downstream targets); and whether the regulation of HH signaling is common in various tissues or strains or species. In this review, we have highlighted the current understanding of the HH signaling pathway and its interactions with other factors and signals. Future studies will be needed to further fine-tune its function and precisely demonstrate the mechanistic role of HH signaling in the regenerative process. These studies could provide new insights regarding previously unknown function of HH signaling which may prove beneficial to promote regeneration.
